# Hepcidin and sports anemia

**DOI:** 10.1186/2045-3701-4-19

**Published:** 2014-04-14

**Authors:** Wei-Na Kong, Guofen Gao, Yan-Zhong Chang

**Affiliations:** 1 Laboratory of Molecular Iron Metabolism, College of Life Science, Hebei Normal University, Shijiazhuang 050016, Hebei Province, P. R. China; 2 Bioreactor and Protein Drug Research and Development Center of Hebei Universities, Hebei Chemical & Pharmaceutical College, Shijiazhuang 050026, Hebei Province, P. R. China

**Keywords:** Iron metabolism, Exercise, Anemia, Hepcidin

## Abstract

Iron is an important mineral element used by the body in a variety of metabolic and physiologic processes. These processes are highly active when the body is undergoing physical exercises. Prevalence of exercise-induced iron deficiency anemia (also known as sports anemia) is notably high in athletic populations, particularly those with heavy training loads. The pathogenesis of sports anemia is closely related to disorders of iron metabolism, and a more comprehensive understanding of the mechanism of iron metabolism in the course of physical exercises could expand ways of treatment and prevention of sports anemia. In recent years, there have been remarkable research advances regarding the molecular mechanisms underlying changes of iron metabolism in response to physical exercises. This review has covered these advances, including effects of exercise on duodenum iron absorption, serum iron status, iron distribution in organs, erythropoiesis, and hepcidin’s function and its regulation. New methods for the treatment of exercise-induced iron deficiency are also discussed.

## Introduction

Iron is an essential trace element in the human body. It is important for the synthesis of hemoglobin and oxygen delivery, and plays a key role in the electron transport chain as well as the production of energy in mitochondria [1,2]. Many of these functions are directly related to physical exercises. There is a high rate in athletes, particularly those with heavy training loads, of being iron deficient, which is resulted from iron losses in hemolysis, hematuria, sweating and gastrointestinal bleeding during sports training [3-6]. Exercise-induced iron deficiency anemia, also known as sports anemia, leads to a decline of athletes’ performances and other physiologic dysfunctions [2,7]. A more comprehensive understanding of the mechanisms of iron deficiency related to sports activities may help improving the methods of diagnoses and treatments of iron related-disorders in athletes.

## Effects of intensive exercise on iron metabolism

Iron metabolism involves three main aspects: iron absorption from the diet in the duodenal enterocytes, iron usage in the erythroid precursors, and iron storage and reutilization in the hepatocytes and tissue macrophages.

### Effects of intensive exercise on intestinal iron absorption

Iron in the diet can be found in two forms, non-heme (inorganic) and heme (organic). Radioactive studies of heme and non-heme iron absorption showed rather low values in athletes involved in intensive physical activities [8]. Studies on rats also found that iron absorption apparently decreased in the strenuously exercised rats as compared to the sedentary controls [9]. However, the molecular mechanisms of regulation on the altered iron absorption during exercise remained unclear. Over the past decade, the findings of several important molecules involved in iron homeostasis, including divalent metal transporter 1 (DMT1), ferroportin1 (FPN1), heme-carrier protein 1 (HCP1), hephaestin (HP) and ceruloplasmin (Cp), have helped to illuminate the mechanism of decreased iron absorption in athletes.

**Figure 1:**
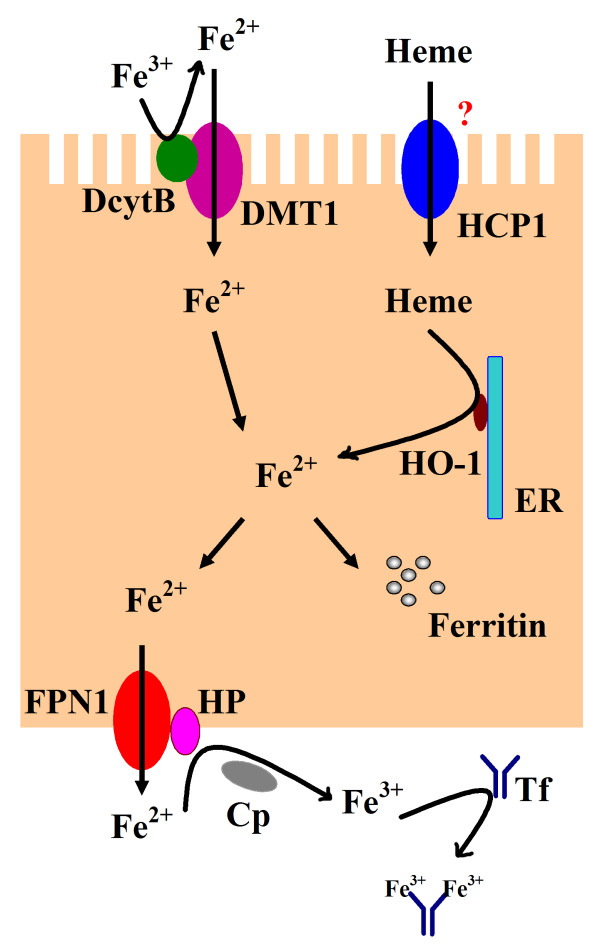
**Schematic of intestinal iron uptake.** Fe^3+^ in the intestinal lumen is converted into Fe^2+^ by DcytB, and Fe^2+^ is then transported across the apical membrane of enterocytes by the transmembrane transporter DMT1. Dietary heme can also be transported across the apical membrane by a yet unknown mechanism. HCP1 is a putative heme transporter that transports heme across the apical membrane of enterocytes into the cytosol. Heme is subsequently metabolized in the cytosol by HO-1 (localized at endoplasmic reticulum membrane facing cytosol) to liberate Fe^2+^. Iron is then stored in ferritin or eventually exported across the basolateral membrane into the bloodstream via Fe^2+^ transporter FPN1. The FPN1-mediated efflux of Fe^2+^ is coupled by its re-oxidation to Fe^3+^, catalysis by the membrane-bound ferroxidase HP, and possibly also by its plasma homologue Cp.

Intestinal iron absorption is a tightly regulated process (Figure 1). Inorganic Fe^3+^ cannot be absorbed well, and duodenal cytochrome B561 (DcytB), a heme-containing enzyme at the apical surface of the enterocytes, converts Fe^3+^ into Fe^2+^ for better absorption [10]. Fe^2+^ is transported across the apical membrane of enterocytes by the transmembrane transporter DMT1, which is also known as DCT1 or Nramp2 [11]. Different combinations of the alternative 5′ and 3′ exons (1A or 1B and IRE or non-IRE, respectively) of DMT1 gene can specify up to four distinct mRNAs encoding four different DMT1 [12]. All these DMT1 isoforms function as iron transporters with equivalent transport efficiency [13]. The mechanism of heme iron absorption is still not well understood. It has been suggested that HCP1, a high-affinity folate influx transporter, is responsible for the uptake of heme into the gut cells from the intestinal lumen [14]. Then heme is catabolized in enterocytes by heme oxygenase-1 (HO-1), producing free iron, biliverdin and carbon monoxide [15]. Iron is then stored in ferritin [16] or transferred out across the basolateral membrane by an integral membrane protein FPN1 [17] in cooperation with HP [18], and possibly also involves its plasma homologue Cp [19]. Our recent studies found that the expression of duodenal DMT1, HCP1 and FPN1 decreased in strenuously exercised rats as compared to the control group [20]. This might be the reason why iron absorption, including both non-heme and heme iron, is reduced in athletes after intensive exercise.

### Effects of intensive exercise on iron usage by erythroid precursors

Erythroid cells are the major iron-utilizing cells in the body. It is generally believed that transferrin and transferrin receptor (TfR) mediated iron delivery is the main pathway of iron uptake in erythroid cells [21,22]. The number of TfR on cell membrane is an important factor to reflect the cell’s ability to uptake iron from transferrin. In addition, a soluble form of the TfR (sTfR) has been identified in animal and human serums. sTfR is released by erythroblasts [23] and reticulocytes [24], and has been established as a quantitative marker of cellular TfR. Levels of TfR on cell membrane and sTfR in serum are both considered as indicators for the marrow’s erythropoietic activity.

Studies by Qian *et al*. [25] found that the average number of surface TfR on erythroblasts significantly increased in the strenuously exercised rats as compared to the controls. A significant increase of the rate of radioactive iron uptake by the erythroblasts of the exercised rats was also observed. The sTfR levels significantly increased as well in the exercised rats when compared with the sedentary group [26]. These results implied that intensive exercise could lead to an increase in erythropoietic activity of the marrow.

### Effects of intensive exercise on iron storage and reutilization

Most of the body iron is stored in parenchymal cells and macrophages of the liver. When the body iron demand increases, iron storage in organs and tissues decreases. Intensive exercise can lead to an increase in erythropoietic activity of the marrow [25,26], and the synthesis of hemoglobin requires a large amount of iron. Therefore, intensive exercise could induce a reduction of iron stores in liver. Studies of Liu *et al*. [20] and Qian *et al*. [25] confirmed the above hypothesis. They found that exercise induced a significant decrease of the iron concentration in liver. However, other reports showed that hepatic iron level increased significantly with acute exercise [27,28]. The contradictory results could come from different training methods, exercise intensity, or length. Hemolysis has been reported to occur in a variety of exercises including swimming, cycling and running [29,30]. When erythrocyte destruction occurs intravascularly, it results in release of hemoglobin into the bloodstream [31]. Then the free hemoglobin is rapidly bound by haptoglobin and cleared from the circulation by parenchymal cells or macrophages of the liver [32]. It has been observed that the haptoglobin level in serum decreased after exercise [31]. Therefore, some of iron is redistributed from red blood cells to the liver due to hemolysis, which might explain why in some studies an increased hepatic iron level resulted from intensive exercises could be observed.

## Sports anemia and hepcidin

Although exercise can increase erythropoietic activity in marrow [25], it was found that erythrocyte numbers, haemoglobin levels and haematocrit values were significantly decreased after intensive training [20,26]. This observation may be resulted from the reduced iron absorption in small intestine and the decreased iron export to the circulation from parenchymal cells and macrophages. Many studies have reported that significant decreases in a series of serum iron status indicators, including serum iron, transferrin saturation and serum ferritin, were observed in both human and animal models following intensive training [20,33-35]. This suggested that the amount of serum iron transferred to bone marrow was reduced after extensive exercise, which may cause the iron concentration in bone marrow cannot meet the demand of the accelerated erythropoietic process. As a result, the increased loss of red blood cells and the insufficient production of new red blood cells together would lead to the occurrence of anemia. However, the regulatory mechanism in iron metabolism that leads to the decreased iron absorption in small intestine and the trapping of iron in liver are unknown. Hepcidin, the principal iron-regulatory hormone responsible for the maintenance of iron homeostasis, controls the absorption of dietary iron and the distribution of iron among organs and tissues in the body [36]. Therefore, the decreased iron absorption and increased hepatic iron stores observed in the exercised rats may closely relate to the hepatic hepcidin expression.

### Effects of intensive exercise on the expression of hepcidin

In recent years, many studies on the function of hepcidin in sports anemia have been reported. A 72 h timeline of hepcidin expression post-exercise was assessed, which showed that the hepcidin level significantly elevated at 3, 6 and 24 h post-exercise, and then declined from there, reaching baseline at 72 h post-exercise [37,38]. These results revealed the association of hepcidin expression with the intensive exercise. Instead of using a single exercise stimulus, studies from our laboratory detected the accumulative effects of intensive exercise on hepcidin expression by having the rats take strenuous treadmill running for 5 weeks. Our results showed that the hepatic hepcidin mRNA increased significantly in the exercised rats, and those rats were diagnosed as sports anemia after 5 weeks of intensive exercise [20]. These findings were consistent with the results observed in human [39].

### Hepcidin causes the reduction of duodenal iron absorption and trapping of intracellular iron during exercise

Nemeth *et al*. [40] have demonstrated that hepcidin can decrease the functional activity of FPN1 by binding to it directly, resulting in its internalization and degradation, and thereby blocking cellular iron efflux. It was also demonstrated that the triggering of FPN1 degradation by hepcidin in hepatocytes could lead to decreased iron export and increased retention of cellular iron [41]. Treatment of macrophages with hepcidin dramatically decreased the FPN1 protein level and reduced the efflux of iron after erythrophagocytosis [42,43]. Administration of His-tagged recombinant hepcidin resulted in significant reduction of duodenal FPN1 expression in rats [44]. Therefore, it can be concluded that hepcidin limits the release of iron from hepatocytes, macrophages and enterocytes by decreasing FPN1 expression and increasing its degradation. In addition, both *in vitro* and *in vivo* studies have demonstrated that the duodenal DMT1 level decreases following hepcidin treatment [45,46]. Further studies showed that the decreased expression of DMT1 by hepcidin was caused by ubiquitin-dependent proteasome degradation in Caco-2 cells (a human intestinal cell line) [47]. These results suggest that hepcidin not only controls iron release to the circulation, but also regulates iron absorption in the intestine.

Taken together, it can be inferred that the increased hepcidin expression after exercise results in the degradation of iron transporters such as DMT1 and FPN1, causing the reduction of iron absorption from small intestine and the trapping of iron in hepatocytes and macrophages. Therefore, it is very likely that the frequently observed iron deficiency in athletes is caused, at least in part, by the elevated hepcidin level. Some of the above speculations have been investigated in our previous studies. We found that hepatic hepcidin mRNA significantly increased in rats trained with strenuous exercise, which was associated with an obvious decrease in duodenal DMT1 and FPN1 expression [20]. However, whether the expression of FPN1 in parenchymal cells or macrophages of the liver alters during exercise remains to be investigated in the future studies.

## Hepcidin expression in moderate exercise

It is well known that strenuous exercise usually leads to the development of sports anemia. In contrast, regular and moderate exercise training might be a promising, safe and economical method to help improve body iron status. Our previous results showed that the levels of serum iron and transferrin saturation in moderately exercised rats were significantly higher than that of the controls [48]. This in turn induced an increased iron transport from blood to bone marrow to synthesize hemoglobin and erythrocytes, resulting in enhanced oxygen-carrying capacity [48].

Studies also investigated the changes of hepcidin expression level during moderate exercise, and found that the moderate exercise did not induce but decreased hepcidin expression [48,49]. Studies further demonstrated that the expression of DMT1 and FPN1 in duodenum of the moderately exercised rats increased significantly as compared to the controls, suggesting that moderate exercise may increase duodenal iron absorption [48]. These results, from a different aspect, imply that hepcidin plays an important role during exercise.

## Hepcidin regulation by exercise

**Figure 2:**
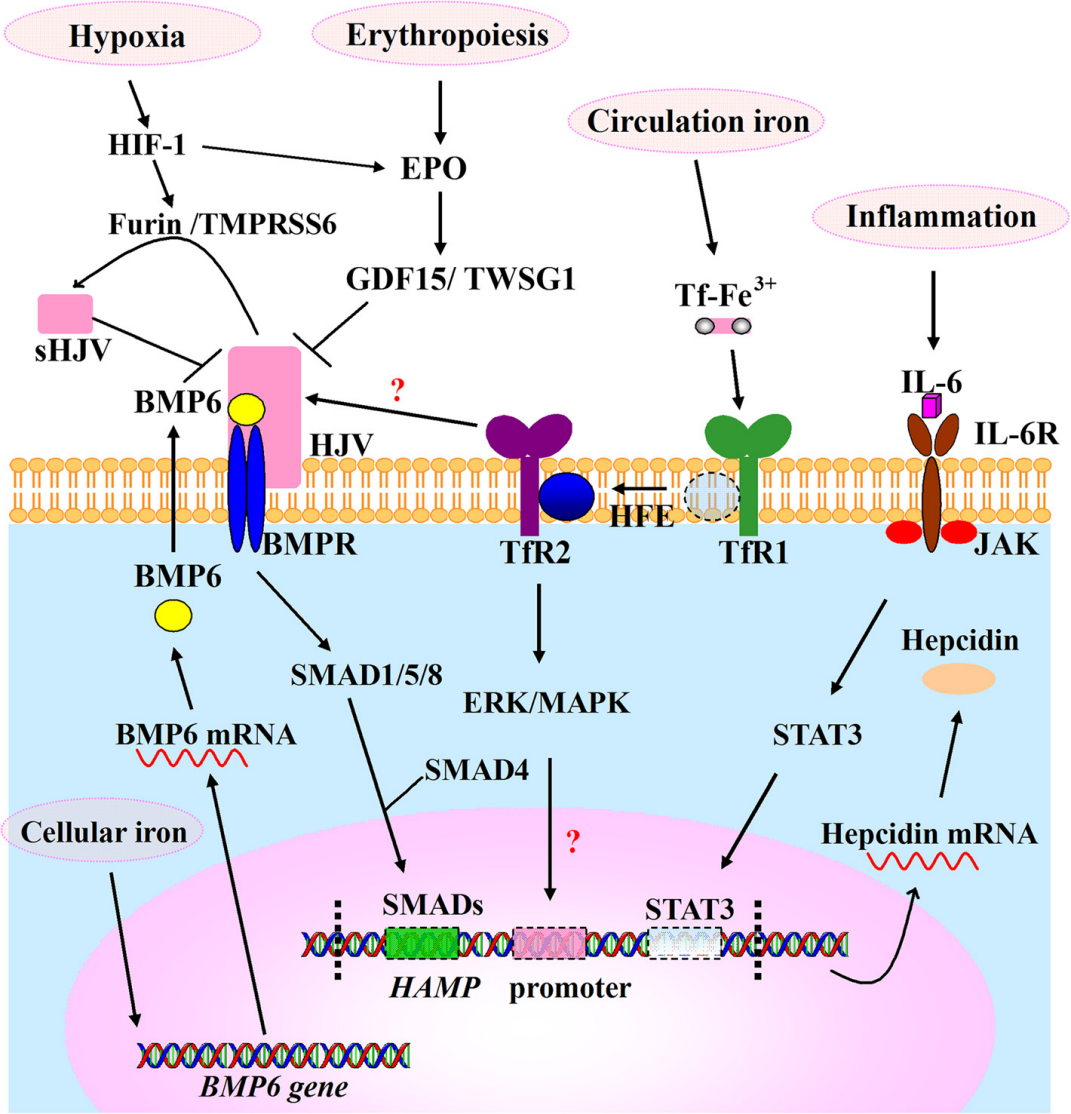
**Molecular mechanisms of hepcidin regulation.** So far, three molecular pathways are found to be involved in the regulation of hepcidin, JAK/STAT3, BMP/SMAD and HFE/TfR2 pathways. Inflammatory stimuli, such as IL-6, induce hepcidin synthesis through the JAK/STAT3 pathway. Hepatic cellular iron can increase the expression of BMP-6. The released BMP6 then interacts with BMPR and HJV to form a complex and activates the SMAD pathway. The SMAD pathway involves phosphorylation of SMAD1, 5, and 8 (pSMADs), formation of pSMADs/SMAD4 complex, and the subsequent translocation of this complex to the nucleus to activate the expression of the hepcidin gene. Extracellular Tf-Fe^2+^ mediates a second iron signal. When the serum transferrin saturation increases, Tf-Fe^2+^ displaces HFE from TfR1. HFE then interacts with TfR2 to form the HFE/TfR2 complex. The HFE/TfR2 complex activates hepcidin transcription via HJV/BMP/SMAD and/or ERK/MAPK signaling pathway. Furthermore, HJV is subjected to cleavage by furin and TMPRSS6 to form a soluble HJV (sHJV), which can selectively inhibit BMP-induced hepcidin expression. Furin and TMPRSS6 can be regulated by hypoxia via HIF-1. Erythropoiesis may control hepcidin expression by EPO production. EPO subsequently stimulates GDF15 expression, which acts together with TWSG1 to inhibit hepatic hepcidin expression by inhibiting the BMP/SMAD pathway.

Hepcidin is produced primarily by hepatocytes. Other tissues and cells, such as macrophages, have been shown to express hepcidin as well, though at a much lower level [50]. So far, it has been demonstrated that hepcidin expression can be influenced by inflammation [51], body iron status [52], erythropoiesis [53], as well as hypoxia [54] (Figure 2).

### Hepcidin regulation by inflammation during exercise

A variety of studies have demonstrated that exercise induces notable physiological changes in the immune system [55]. Strenuous exercise can induce a dramatic increase in the levels of pro-inflammatory cytokines and inflammation-responsive cytokines [56]. Interleukin-6 (IL-6) is the major cytokine that was produced at a significant higher amount in response to exercise than other cytokines [57,58], while the contracting muscles contribute to most of the IL-6 production in the circulation in response to exercise [57,59].

Hepcidin synthesis was markedly induced by infection and inflammation [51,60], and IL-6 itself is sufficient to induce hepcidin expression during inflammation [61]. Significant higher amount of hepatic hepcidin mRNA was detected 3 to 6 h after IL-6 stimulation, which indicates that the production of IL-6 in contracting skeletal muscles results in the exercise-induced hepcidin increase. Studies by Peeling *et al*. [38] support this hypothesis as it was found that hepcidin levels elevated 3 h after the peak production of IL-6 induced by exercise. In addition, animals treated with cyclosporin A, a calcineurin inhibitor to blunt plasma IL-6 during exercise, showed lower hepcidin levels as compared to the exercised group without cyclosporin A treatment [62]. These results suggest that IL-6 is involved in the exercise-induced increase of hepcidin expression. Furthermore, it has been demonstrated that the stimulatory effect of IL-6 on hepcidin expression exhibits at the transcriptional level. Hepcidin expression is directly induced by IL-6 through the activation of the janus kinase/signal transducer and activator of transcription-3 (JAK/STAT3) signaling pathway during inflammatory stimulation [63-65].

### Hepcidin regulation by iron status during exercise

How iron regulates the expression of hepcidin has been a hot area of study in this field. By studying the naturally occurring mutations in humans and using transgenic mouse models, valuable information has been obtained regarding the key molecules involved in the regulation of hepcidin expression by circulating iron and hepatic stored iron. These molecules include HFE (HLA2-linked hemochromatosis gene) [66,67], transferrin receptor 2 (TfR2) [68-70], hemojuvelin (HJV) [71] and bone morphogenetic protein (BMP) [72]. A possible model on the regulation of hepcidin by iron is proposed based on these results (Figure 2). Briefly, hepcidin expression is regulated by hepatic cellular iron stores through Bone morphogenetic protein 6 (BMP6) signaling. BMP6 is an activating ligand for BMP receptor (BMPR), and its level reflects the level of hepatic iron stores. When liver iron concentration is high, the production of hepatocyte BMP6 is increased. Then, the released BMP6 from the hepatocyte forms a complex with BMPR and HJV. Binding of BMP6 to BMPR controls the transcription of hepcidin by activating the SMAD pathway. The liver uses transferrin saturation as an extracellular iron sensor. When the serum transferrin saturation increases, HFE is dislodged from its binding site on TfR1, and then interacts with TfR2 to form the HFE/TfR2 complex. This complex participates in the hepcidin regulation though activation of the extracellular signal-regulated kinases/the mitogen activated protein kinase (ERK/MAPK) pathway and/or HJV/BMP/SMAD pathway [36,52,73-76].

HJV plays a central role in the regulation of hepatic hepcidin expression. HJV acts as a coreceptor for BMP to increase the sensitivity of BMPR to BMP, and various hepcidin regulatory pathways seem to be converged at this protein (Figure 2). Recent researches showed that HJV is not only highly expressed in liver, but also in skeletal muscles. Studies from our laboratory found the levels of HJV mRNA in liver and skeletal muscle were remarkably higher in the exercised rats than in the controls [20]. Thus, hepcidin expression may also be induced by the elevation of HJV during exercise. However, the underlying mechanism of HJV-regulated hepcidin expression in response to sport remains to be explored.

### Hepcidin regulation by erythropoiesis during exercise

Studies by Qian *et al.*[25] and Tian *et al.*[26] both indicated that extensive exercise could lead to an increase in erythropoietic activity of bone marrow. It has also been demonstrated that increased erythropoiesis can suppress hepcidin expression significantly [51]. However, the relationship between increased erythropoiesis and decreased hepcidin expression and the molecules involved remain unclear. Erythropoietin (EPO), an endogenous hormone produced primarily by kidney, is a key regulator of erythropoiesis. EPO promotes the proliferation and differentiation of the erythroid progenitor cells [77]. Increases in serum EPO concentration have been observed in athletes [78], and many studies also reported that hepatic hepcidin expression strongly reduced by EPO treatment [79,80]. Therefore, EPO has been considered to be a potential mediator of hepcidin regulation. The *in vitro* studies by Pinto *et al.*[81] indicated that the EPO suppression on hepcidin expression was achieved by modulating the C/EBPα mRNA and decreasing its protein levels, thereby resulting in significant changes in the transcription of hepcidin mRNA. However, this hypothesis has not been confirmed *in vivo*. Studies in animal models found that the suppression of hepcidin expression by EPO administration could be recovered by using the inhibitors of erythropoiesis via irradiation or posttransfusion polycythemia [82,83]. These results suggest that EPO decreases hepcidin transcription only when the erythropoiesis is active. Therefore, the suppression of hepcidin expression was not directly mediated by EPO, and other erythropoietic factors may be involved.

Growth differentiation factor 15 (GDF15), a member of the transforming growth factor-beta superfamily, is produced by erythroid cells during erythroblast maturation to promote differentiation of erythroid [84]. It has been found that GDF15 significantly increased immediately after a 246-km foot-race, and then it showed a decline at 48 h post-exercise, but was still above the baseline level. Erythroid-specific production of GDF15 is fully dependent on EPO stimulation [85]. Therefore, GDF15 might provide a link between erythropoiesis and hepatic hepcidin regulation. In fact, GDF15 has been identified as a hepcidin-suppression factor that expresses at high levels in thalassemia patients with ineffective erythropoiesis. In cultured hepatocytes, high level of GDF15 has also been found to suppress hepcidin expression [86]. Twisted gastrulation (TWSG1), an erythroid signaling molecule that expresses at early stages during erythropoiesis, was proposed to act together with GDF15 to inhibit hepatic hepcidin expression through inhibiting the BMP-dependent activation of SMAD-mediated signal transduction [87]. However, this proposed mechanism is still controversial. Casanovas et al. [88] found that hepatic hepcidin mRNA expression was not altered in GDF15^−/−^ mice under steady state conditions or upon phlebotomy as compared to wild-type mice, which suggests that GDF15 is not involved in the down-regulation of hepcidin under steady state conditions or in response to blood loss in mice. Therefore, whether GDF15 is involved in the hepcidin regulation by erythropoiesis during exercise needs to be further studied.

### Hepcidin regulation by hypoxia during exercise

Many studies have demonstrated that training athletes at high altitude may significantly increase their VO_2_max and RBC mass and thereby improve their endurance performance [89,90]. Further studies found that the adaptation in iron metabolism will appear under high altitude and hypoxia conditions in mountaineers. Under hypoxia conditions, serum duodenal DMT1 and FPN1 mRNA increased and hepcidin level decreased. These changes would result in increased dietary iron uptake and iron release from iron stores to ensure a sufficient iron supply for hypoxia-induced erythropoiesis [91].

The mechanism of hepcidin suppression by the hypoxia at high altitude is also unclear. It is likely that hypoxia-inducible transcription factor (HIF), the master regulator of the systemic and cellular adaptation to hypoxia, plays a role in hepcidin regulation. Peyssonnaux *et al.*[92] proposed that HIF regulates hepcidin expression directly via transcriptional suppression. However, this could not be confirmed in the isolated hepatocytes [93], and other indirect pathways by which HIF regulates hepcidin expression may exist. It has been found that HIF could regulate renal and hepatic EPO synthesis directly under hypoxia [94], and the activation of hepatic HIF itself without the concomitant increase in EPO transcription did not suppress hepcidin expression [95]. Therefore, the regulation of HIF on hepcidin may be mediated by affecting the EPO synthesis. Taken together with the above mentioned association between EPO induction and elevated serum GDF15 level [91], it can be inferred that HIF-associated suppression of hepcidin may occur indirectly through EPO-induced erythropoiesis and its subsequent signaling via GDF15 [95]. Furthermore, HIF was reported to induce the production of furin and transmembrane serine proteinase TMPRSS6 (also known as matriptase-2), two proteases that mediate the release of soluble hemojuvelin (s-HJV) by cleaving HJV off from the cell membrane [96,97]. s-HJV was found to competitively inhibit BMP-induced hepcidin expression [98,99]. Therefore, HIF may also suppress hepcidin expression by increasing the breakdown of HJV and inhibiting the BMP-induced hepcidin expression.

### Hepcidin in the premenopausal female athletes

It is believed that female athletes may experience higher risk of sports anemia than male athletes due to the iron losses in some physiologic processes, such as menstruation [100]. However, it has been found that erythropoiesis induced by blood loss could lead to a decrease in hepcidin expression, thereby increasing iron level in the body. By using the serum enzyme-linked immunosorbent assay, it was found that healthy women had lower serum hepcidin levels than healthy men [101]. Animal studies also found that bleeding provoked by repetitive phlebotomies was associated with a dramatic decrease in hepatic hepcidin level [51]. However, the precise mechanism on hepcidin regulation associated with sex differences is not clear. Studies by Ikeda *et al*. showed that the female sex hormone, estrogen, can stimulate hepcidin expression in a GPR30-BMP6-dependent pathway [102]. In contrast, testosterone can suppress hepcidin expression potently via testosterone/AR/SMAD [103] or testosterone/EGF/EGFR signaling pathways [104]. Based on these above results, it puzzles to find that the serum hepcidin level in women is lower than that in men. We hypothesized that physiological loss of blood in pre-menopausal women had a suppressive effect on hepcidin transcription, which may counteract the stimulating effect of estrogen on hepcidin expression to some extent. If menstruation in the female athletes does not exist, they still would develop iron deficiency anemia much rapider than the male athletes because of the high level of hepcidin induced by estrogen.

## Methods to measure the hepcidin level

Because of the important functions of hepcidin in athlete’s iron homeostasis, a reliable assay to measure hepcidin levels in the body fluids of athletes should be developed. However, for many years, it has been difficult to detect hepcidin by the conventional immunochemical assay. This is mainly due to the problems in generating specific anti-hepcidin antibodies in animals because of low immunogenicity of the short hepcidin peptides [79]. Hepcidin is synthesized by the liver in the form of an 84 amino-acid pro-peptide, but it is detected in the plasma as three isoforms including hepcidin-20, -22, and −25 [105]. The hepcidin-20 and −22 isoforms play no role in the regulation of iron metabolism, but they can interfere with the quantification of hepcidin-25, because the antibodies used in the immunoassays can react with all of three hepcidin isoforms.

In recent years, substantial progress has been made in the measurement of hepcidin. These methods can be divided into two main methodologies. The first one is mass spectrometry (MS)-based hepcidin measurement, which has the advantage of distinguishing hepcidin-25, -22, and −20 in various body fluids, including serum and urine samples, but it requires relatively expensive equipment [106-109]. The second method, immunochemical assays including competitive radioimmunoassay (RIA) [110], competitive enzyme-linked immunosorbent assay (ELISA) [107,111] and dual-monoclonal sandwich ELISA [112], is suitable for the large-scale quantification of serum hepcidin because of its low cost and high-throughput features, but these assays usually measure the amount of all three hepcidin isoforms [112].

## Treatment of sports anemia

### The use of iron-supplementary

For iron deficiency anemia, iron supplement therapy is a relatively safe and economical method, but the efficacy of iron supplement is often very low. Previous studies have demonstrated that iron supplementation can lead to the increase of serum ferritin without accompanied by the increase in hemoglobin concentration [113]. On the other hand, increased iron stores in the body are a common finding in elite athletes who have used long-term iron supplementation, putting the athletes at an increasing risk of developing iron overload-related diseases [114]. The combination of exercise-induced decrease of iron absorption in duodenum and increase of iron retention in hepatocytes and macrophages may be responsible for the above observation. Therefore, investigations to explore new methods for the treatment of sports anemia should be initiated.

### The use of hepcidin antagonists

The recent advances in the molecular mechanisms of iron regulation reveal that the elevated hepcidin expression is a key factor in the development of sports anemia. Hepcidin inhibits iron absorption in enterocytes and causes iron sequestration in hepatocytes and macrophages [36]. Therefore, using hepcidin antagonists to modulate hepcidin expression is a novel alternative therapy for the treatment of sports anemia. Although no such therapy has yet been available, several candidates are currently under development.

#### Methods to neutralize hepcidin’s activity

##### Monoclonal antibodies of hepcidin

Cooke *et al.*[115] generated anti-hepcidin human antibodies as a potential therapeutic for the treatment of anemia of inflammation (AI), which has the similar iron-related disorders to sports anemia. They found that these antibodies can increase the availability of serum iron and lead to enhanced red cell hemoglobinization both in mouse and cynomolgus monkey models of AI. However, hepcidin antibodies may reduce the natural clearance of hepcidin in the circulation and thus result in further hepcidin accumulation.

##### Small-molecule antagonists of hepcidin

Fung *et al.*[116] identified a small molecule, named fursultiamine, which inhibits the hepcidin-FPN1 interaction. They found that fursultiamine directly interfered with hepcidin binding to FPN1, thereby preventing hepcidin-induced FPN1 ubiquitination, endocytosis and degradation. This allowed continuous cellular iron export despite of the presence of hepcidin. Studies by Schwoebel *et al.*[117] found another anti-hepcidin compound, NOX-H94, which can also protect FPN1 from hepcidin-induced degradation.

#### Methods to decrease hepcidin expression

In addition to directly interfering with hepcidin activity, therapies can be effective by targeting erythropoiesis pathway, inflammatory pathway, or HJV/BMP/SMAD signaling pathway to decrease the hepcidin production.

##### EPO doping

EPO is the primary signal that triggers erythropoiesis in anemic and hypoxic conditions. Therefore, EPO has been investigated as a drug to increase oxygen transport and improve athletic endurance capacity [118]. EPO has been frequently used by athletes as a performance-enhancing agent [119]. In 1990, the International Olympic Committee (IOC) prohibited the use of EPO in sports. Without considering its legality, EPO doping administration has the potential of correcting some misregulations in iron metabolism of athletes. Previous studies in our lab observed that administration of recombinant human erythropoietin (rHuEPO) induced a dramatic reduction in hepcidin expression, which is closely associated with the increased intestinal iron absorption and macrophage iron release [53,120]. However, athletes, who use EPO to enhance their performance, are taking great health risks. Elevated haematocrit and dehydratation during intense exercise may induce high blood viscosity, which is often associated with high blood cardiovascular problems, such as hypertension, heart hypertrophy and brain vascular congestion [121].

##### Anti-IL6 antibodies and STAT modulators

Anti-cytokine therapeutics, such as anti-IL-6 antibodies, can block the induction of hepcidin and improve anemia [122,123]. AG490, a small molecule of STAT3 inhibitor, can suppress hepcidin transcription and increase serum iron levels in mice by inhibiting the JAK/STAT signaling pathway [124]. CO-releasing molecules can also inhibit the IL-6-induced hepcidin expression through inhibition of the STAT3 pathway [125]. However, the anti-cytokine therapy may lead to an increased risk for severe infections due to the impaired host defense [126].

##### BMP modulators

As mentioned above, s-HJV decreases hepcidin expression by competing with HJV and interfering with BMP signaling [98]. Studies by Theurl *et al*. [127] demonstrated that inhibition of hepcidin expression by using the soluble hemojuvelin-Fc (sHJV.Fc) in a rat model of AI resulted in the mobilization of iron stores, increase of serum iron levels, stimulation of erythropoiesis, and correction of anemia. In addition, inhibition of BMP type I receptor signaling by small molecules, such as dorsomorphin, was also effective in alleviating iron deficiency anemia. Dorsomorphin can block BMP-mediated SMAD1/5/8 phosphorylation, reduce hepcidin expression and raise serum iron levels [128]. Heparin, another potent inhibitor of hepcidin expression, can induce a strong reduction in serum hepcidin both in animal models and humans, which finally led to an increase in serum iron levels [129]. It functions likely by sequestering BMP6 and blocking of SMAD signaling [129]. However, since BMP pathway plays a variety of important functions in the body, the use of BMP modulators may have various adverse effects. BMP modulators as potential drugs to reduce hepcidin expression should have a great specificity.

## Conclusions

Iron is an essential trace element required to support the physical functions in human body. However, iron deficiency is commonly found in strenuous exercised athletes. In addition to those well-known processes of iron loss, such as hemolysis, hematuria, sweating and gastrointestinal bleeding, exercise-induced up-regulation of hepcidin expression might be the main reason that results in iron deficiency in athletes. Taken together of our work with others’ reports, we infer that the increased hepcidin inhibits both brush border iron uptake and basolateral export through the down-regulation of key iron transporters, DMT1 and FPN1. In hepatocytes and macrophages, hepcidin decreases FPN1 expression and results in a decreased iron export and cellular iron retention. Reduced intestinal iron absorption, sequestered iron in hepatocytes and macrophages, and elevated iron demand for erythropoiesis after extensive exercise can lead to a low-iron status, indicated by low serum iron concentration, transferrin saturation and markedly decreased non-heme iron concentration in some tissues. If the amount of iron in marrow could not meet the needs for erythropoiesis during exercise, iron deficiency anemia will be developed. Therefore, iron homeostasis will be destroyed after the prolonged strenuous exercise, which does harm athletes’ performance and health. Molecules IL-6, HJV, EPO and HIF were found to be involved in the exercise-induced alteration of hepcidin expression. The possible regulatory mechanism for each molecule was discussed in this review. However, our current understanding of the detailed mechanisms on exercise-induced alteration of iron metabolism is still incomplete, and further studies are needed. Recent advances regarding the drugs that can decrease hepcidin expression were summarized in this review, which will provide some insights into the development of potential therapeutics for treatment of sports anemia in the future.

## Abbreviations

DMT1: Divalent metal transporter 1; FPN1: Ferroportin1; HCP1: Heme-carrier protein; HP: Hephaestin; Cp: Ceruloplasmin; DcytB: Duodenal cytochrome B561; HO: Heme oxygenase; TfR: Transferring receptor; IL-6: Interleukin-6; JAK: Janus kinase; STAT3: Signal transducer and activator of transcription-3; HJV: Hemojuvelin; BMP: Bone morphogenetic protein; ERK: Extracellular signal-regulated kinase; MAPK: Mitogen activated protein kinase; EPO: Erythropoietin; GDF15: Growth differentiation factor 15; HIF: Hypoxia-inducible transcription factor; TWSG1: Twisted gastrulation; ELISA: Enzyme-linked immunosorbent assay; rHuEPO: Recombinant human erythropoietin; AI: Anemia of inflammation; RIA: Radioimmunoassay.

## Competing interests

The authors declare that they have no competing interests.

## Authors’ contributions

Y-ZC conceived the review and participated in design and discussion. W-NK wrote the main parts of the manuscript and participated in discussion. GG participated in discussion and writing of the manuscript. All authors read and approved the final manuscript.
